# *IFT81*, encoding an IFT-B core protein, as a very rare cause of a ciliopathy phenotype

**DOI:** 10.1136/jmedgenet-2014-102838

**Published:** 2015-08-14

**Authors:** Isabelle Perrault, Jan Halbritter, Jonathan D Porath, Xavier Gérard, Daniela A Braun, Heon Yung Gee, Hanan M Fathy, Sophie Saunier, Valérie Cormier-Daire, Sophie Thomas, Tania Attié-Bitach, Nathalie Boddaert, Michael Taschner, Markus Schueler, Esben Lorentzen, Richard P Lifton, Jennifer A Lawson, Meriem Garfa-Traore, Edgar A Otto, Philippe Bastin, Catherine Caillaud, Josseline Kaplan, Jean-Michel Rozet, Friedhelm Hildebrandt

**Affiliations:** 1 Laboratory of Genetics in Ophthalmology, INSERM UMR 1163, Paris, France; 2 Paris Descartes—Sorbonne Paris Cité University, Imagine Institute, Paris, France; 3 Division of Endocrinology and Nephrology, Department of Internal Medicine, University Clinic Leipzig, Leipzig, Germany; 4 Division of Nephrology, Department of Medicine, Boston Children's Hospital, Harvard Medical School, Boston, Massachusetts, USA; 5 Pediatric Nephrology Unit, University of Alexandria, Alexandria, Egypt; 6 INSERM UMR 1163, Molecular bases of hereditary kidney diseases, Nephronophthisis and Hypodysplasia, Paris, France; 7 INSERM UMR 1163, Molecular and Physiopathological bases of osteochondrodysplasia, Paris, France; 8 INSERM UMR 1163, Embryology and genetics of human malformation, Paris, France; 9 Department of Pediatric Radiology, Hôpital Necker-Enfants Malades, APHP, Descartes University, Paris, France; 10 Department of Structural Cell Biology, Max Planck Institute of Biochemistry, Martinsried, Germany; 11 Department of Genetics, Howard Hughes Medical Institute, Yale University School of Medicine, New Haven, USA; 12 INSERM UMR 1163, Cell imaging platform, Paris, France; 13 Departments of Pediatrics, University of Michigan, Ann Arbor, USA; 14 Trypanosome Cell Biology Unit, Institut Pasteur and CNRS, URA 2581, Paris, France; 15 Biochemistry Department, Necker Hospital, Paris, France; 16 Howard Hughes Medical Institute, Chevy Chase, Maryland, USA

**Keywords:** Genetics, Molecular genetics, Ophthalmology, Renal Medicine

## Abstract

**Background:**

Bidirectional intraflagellar transport (IFT) consists of two major protein complexes, IFT-A and IFT-B. In contrast to the IFT-B complex, all components of IFT-A have recently been linked to human ciliopathies when defective. We therefore hypothesised that mutations in additional IFT-B encoding genes can be found in patients with multisystemic ciliopathies.

**Methods:**

We screened 1628 individuals with reno-ocular ciliopathies by targeted next-generation sequencing of ciliary candidate genes, including all IFT-B encoding genes.

**Results:**

Consequently, we identified a homozygous mutation in *IFT81* affecting an obligatory donor splice site in an individual with nephronophthisis and polydactyly. Further, we detected a loss-of-stop mutation with extension of the deduced protein by 10 amino acids in an individual with neuronal ceroid lipofuscinosis-1. This proband presented with retinal dystrophy and brain lesions including cerebellar atrophy, a phenotype to which the *IFT81* variant might contribute. Cultured fibroblasts of this latter affected individual showed a significant decrease in ciliated cell abundance compared with controls and increased expression of the transcription factor *GLI2* suggesting deranged sonic hedgehog signalling.

**Conclusions:**

This work describes identification of mutations of *IFT81* in individuals with symptoms consistent with the clinical spectrum of ciliopathies. It might represent the rare case of a core IFT-B complex protein found associated with human disease. Our data further suggest that defects in the IFT-B core are an exceedingly rare finding, probably due to its indispensable role for ciliary assembly in development.

## Introduction

Intraflagellar transport (IFT) is an ancient kinesin and dynein-mediated bidirectional trafficking system essential for cilium assembly and maintenance. It has been conserved from green algae (*Chlamydomonas reinhardtii*) to humans. There are two major IFT subcomplexes within the cilium, A and B. While the IFT-A complex is primarily involved in retrograde transport from tip to base, the IFT-B complex is mainly part of the anterograde transport from base to tip.^1^ Very recently, all six components of the IFT-A complex have been found defective in individuals with a distinct form of nephronophthisis-related ciliopathies (NPHP-RC), namely skeletal ciliopathies.^2^ Skeletal ciliopathies primarily present with a bone-related phenotype such as shortened long bones and ribs (eg, in short rib-polydactyly syndromes, MIM 263510; Jeune asphyxiating thoracic dystrophy, MIM 208500), phalangeal cone-shaped epiphyses (eg, in Mainzer-Saldino syndrome, MIM 266920) or dolichocephaly and hypo/microdontia (eg, in Sensenbrenner syndrome/cranioectodermal dysplasia; MIM 218330).^2^

With regards to the IFT-B complex, defects in only 4 out of 14 members have been associated with human disease to date. Those four components are IFT80, IFT88, IFT172 and very recently IFT27.^3–6^ Defects in any of them result in short rib-polydactyly syndromes, Jeune asphyxiating thoracic dystrophy, Mainzer-Saldino syndrome, or a reno-oculo-hepatic ciliopathy with polydactyly (Bardet-Biedl syndrome, MIM 209900), in the case of IFT27. One of the possible reasons why the IFT-B complex has not been as commonly associated with human ciliopathy phenotypes as the IFT-A complex, is its crucial role in ciliogenesis, as evidenced by embryonic lethality in many established mutant mouse models.^7^ ^8^ The IFT-B complex consists of a nine-subunit salt-stable core (IFT88, IFT81, IFT74, IFT70, IFT52, IFT46, IFT27, IFT25, IFT22/RABL5) and five peripheral components (IFT172, IFT80, IFT57, IFT54/TRAF3IP1, IFT20). Peripheral components are characterised by dissociation from the core at NaCl concentrations above 300 mM.^9^ Although first described in *Chlamydomonas*, a very similar subcomplex composition was also characterised in mice.^10^ Interestingly, the previously identified IFT-B complex components associated with ciliopathies in humans, *IFT80* (MIM 611177) and *IFT172* (MIM 607386), encode for non-core units, whereas defects of core members have only been found in a single case with an evolutionary conserved missense mutation in *IFT88* and in a single case in *IFT27* encoding a small GTPase.^5^ ^6^ We therefore investigated in a large cohort of 1628 individuals with NPHP-RC, retinal ciliopathies, or multisystemic ciliopathies whether recessive mutations in genes encoding for IFT-B core components could be detected.

## Methods

### Patients

Written informed consent was obtained from 1628 individuals with reno-ocular ciliopathies (1056 patients at University of Michigan/Boston Children’s Hospital with NPHP-RC + 572 patients with either NPHP-RC (n=202), skeletal ciliopathies (n=158), embryonically lethal ciliopathies (n=136), early onset retinal dystrophies without cerebro-skeleto-renal symptoms (n=69), and oculo-cerebro ciliopathies (n=7) at INSERM UMR1163, *Imagine Institute of genetic diseases*, Paris). The diagnosis of early onset severe retinal dystrophy and NPHP-RC was based on published clinical criteria.^11^ ^12^

### Targeted and whole exome resequencing

Genomic DNA was extracted from peripheral blood samples by standard salt precipitation methods.

Targeted amplification in the Boston cohort was performed by multiplexed PCR using Fluidigm Access-Array technology followed by barcoding and next-generation resequencing (NGS) on an Illumina MiSeq platform, as previously established by our group.^13^ ^14^ Sanger DNA sequencing was further conducted for single mutation confirmation. All coding exons and adjacent splice sites of the following 14 IFT-B encoding genes were screened: *IFT172/SLB, IFT88*, *IFT81/CDV1, IFT80, IFT54/TRAF3IP1, IFT22/RABL5, IFT52, IFT46, IFT57/HIPPI, IFT74/CCDC2, HSPB11/IFT25, IFT20, IFT27, TTC30B/IFT70* (see online supplementary table S1). In the Paris cohort, ‘ciliome resequencing’ was carried out by a 5.3 Mb customised Agilent Sureselect Target Enrichment Library capturing 32 146 exons of 1666 genes selected from CiliaProteome, Ciliadb and data from the literature.^15^ In individual A3286-21, we subsequently combined homozygosity mapping and whole exome resequencing (WER) for complete mutation analysis. For genome-wide homozygosity mapping, the ‘Human Mapping 250 k StyI’ array was used. Genomic DNA samples were hybridised, and scanned using the manufacturer's standard protocol at the University of Michigan core facility. Non-parametrical logarithm of odds scores were calculated using a modified version of the programme GENEHUNTER V.2.1^16^ through stepwise use of a sliding window with sets of 110 SNPs using the programme ALLEGRO.^17^ Genetic regions of homozygosity by descent (‘homozygosity peaks’) were plotted across the genome as candidate regions for recessive genes, as described in Gee *et al*.^18^ Disease allele frequency was set at 0.0001, and Caucasian marker allele frequencies were used. WER in A3286-21 and variant burden analysis was performed as described previously.^19^ In brief, genomic DNA was isolated from blood lymphocytes and subjected to exome capture using Agilent SureSelect human exome capture arrays (Life technologies) followed by NGS on the Illumina sequencing platform as previously described. Illumina's processing software ELAND (CASAVA V.1.8.2) was used to map reads to the human reference genome (build 19), and SAMtools37 was used to call single nucleotide variants and insertion/deletion at targeted bases. Variants with minor allele frequencies <1% in the Yale (1972 European subjects), National Heart Lung and Blood Institute Grant Opportunity (NHLBI) GO Exome Sequencing Project (4300 European and 2202 African American subjects; last accessed November 2012), dbSNP (V.135) or 1000 Genomes (1094 subjects of various ethnicities; May 2011 data release) databases were selected and annotated for impact on the encoded protein and for conservation of the reference base and amino acid among orthologs across phylogeny. The whole exome of NCK033 was captured using the SureSelect Human All Exon Kits V.3 (Agilent, France). Ciliome and exome were sequenced (2×75 bp) using the Illumina HiSeq2000 system at the Genomic Core Facility of the Imagine Institute (Paris, France). Sequences were aligned to the human genome reference sequence (hg19 assembly), and SNPs were called based on allele calls and read depth using the CASAVA pipeline (Consensus Assessment of Sequence and Variation 1.8, Illumina). Genetic variation annotation was performed by an inhouse pipeline. Only the variants whose positions were covered ≥10× were further considered. Applied exclusion criteria further comprised (1) synonymous or intronic variants other than those affecting the consensus splice sites; (2) variants seen in more than 1% of an inhouse exome data set (n=5571) from unrelated projects; and (3) variants with a minor allele frequency greater than 0.5% in either the 1000 genomes or the exome variant server (EVS) data sets. We hypothesised an autosomal recessive mode of inheritance and focused our attention on homozygous variants.

### Reverse transcriptase PCR

Total RNA was extracted using the RNeasy Mini Kit (Qiagen, France) according to the manufacturer’s protocol. All samples were DNase treated by the RNase-free DNase set (Qiagen). Concentration and purity of total RNA was assessed using the Nanodrop-8000 spectrophotometer (Thermo Fisher Scientific, France). First-stranded cDNA synthesis was performed from 500 ng of total RNA extracted using Verso cDNA kit (Thermo Fisher Scientific) with random hexamer:anchored oligo (dT) primers at a 3:1 (vol:vol) ratio according to the manufacturer’s instructions. A non-reverse transcriptase (RT) reaction (without enzyme) for one sample was prepared to serve as control in RT-qPCR experiments.

### Quantitative real-time RT-PCR

Patient (n=1) and control (n=3) fibroblasts were serum-starved for 48 h, and either exposed to a smoothened agonist (SAG, 100 nM) or negative control for 24 h. RNA was extracted separately for each condition and converted into cDNA. cDNAs were amplified as 161, 156, 103 and 140 bp fragments using specific primers designed from the GLI1NM_005269.2, GLI2NM_005270.4, SMONM_005631.4 and PTCH1NM_000264.3 sequences, respectively (see online supplementary table S2). A 99 bp fragment of the human albumin gene (ALB, NM_000477) was used to control the non-contamination of cDNAs by genomic DNA. TBP, B2M, GUSB, HPRT1, RPLP0 and ALB primers have been previously reported.^20^ cDNAs (5 µL of a 1:25 dilution in nuclease-free water) were subjected to real-time PCR amplification in a buffer (20 µL) containing SYBR® Green Master mix (Applied Biosystems) and 300 nmol/L of forward and reverse primers, on a mastercycler realplex2 (Eppendorf). Data were analysed using the realplex software (Eppendorf). For each cDNA sample, the mean of quantification cycle (Cq) values was calculated from triplicates (SD<0.5 Cq). *GLI1, GLI2, SMO* and *PTCH1* expression levels were normalised to the ‘normalisation factor’ obtained from the geNorm software for Microsoft Excel^21^ which uses the most stable reference genes and amplification efficiency estimates calculated for each primer pair using fourfold serial dilution curves (1:5, 1:25, 1:125, 1:625). Absence of amplification when using mRNA (non-RT) and water (W) as templates, and non-contamination of cDNAs by genomic DNA (ALBh) were controlled in each run (Cq values W=undetermined, non-RT >40 and ALBh >40).The quantitative data are the means±SEM of three independent experiments and these are presented as ratio among values for individual mRNAs. The significance of variations among samples was estimated using the protected least significant difference of Fisher according to the significance of analysis of variance test (Statview Software, V.5; SAS Institute, Cary, North Carolina, USA).

### Cilia abundance and ciliary length measurements

Serum-starved primary cultured fibroblasts from four human controls and patient (NCK033) were fixed with methanol 100% and blocked with bovine serum albumin (BSA) 3% and triton 0.1% in phosphate buffered saline (PBS). Ciliary axoneme and basal bodies were stained overnight at 4°C using mouse monoclonal antiacetylated α-tubulin (Sigma Aldrich; 1:1000) and rabbit polyclonal antipericentrin (1:1000, Abcam) antibodies, respectively. Primary antibodies were labelled for 1 h at room temperature using AlexaFluor 594 goat antimouse (Molecular probe; 1:1000) and AlexaFluor 488 goat antirabbit (1:1000 Invitrogene) secondary antibodies. Images were recorded from Zeiss LSM700 microscope (×40 magnification, Carl Zeiss). Mean numbers of ciliated cells were calculated from 973 patient cells and 895 cells from four individual controls, in two independent experiments. Cilia lengths were measured from the same immunofluorescent images. Mean numbers of cilia <3 μm and >3 μm in length were determined from 100 and 150 patient and control cells, respectively. Data from patient and control cells were compared using the Protected Least Significant Difference of Fisher (PLSD) of Fischer according to the significance of the Student's t test.

### Immunofluorescence analysis of IFT81 cilia localisation

Patient and control fibroblasts were cultured and prepared as described previously. IFT81 was stained overnight at 4°C with, centrioles, ciliary axoneme or subdistal appendages using rabbit polyclonal anti-IFT81 (1:200, Proteintech), goat monoclonal γ-tubulin (1:200; Santa Cruz), mouse monoclonal antiacetylated α-tubulin (1:1000; Sigma Aldrich) or mouse monoclonal anti-ODF2 (1:100, Novus) antibodies. Secondary antibodies were used as described above. Images were recorded from a Gated STimulated Emission Depletion (STED) Leica SP8. The intensity of IFT81 staining at the cilia base and at the tip was quantified by using imageJ software. Average fluorescent intensities were determined from the region of interest drawn around the cilium from maximum intensity Z projection images. Centriolar and tip intensities and ratio of one to the other were compared in patient and control cell lines.

### Immunofluorescence staining of IFT22, IFT25, IFT46, IFT88, GLI1, GLI2 and SMO

Patient and control fibroblasts were prepared as described previously. Ciliary axoneme and IFT22, IFT25, IFT46, IFT88, GLI1, GLI2 or SMO were stained overnight at 4°C using mouse monoclonal antiacetylated α-tubulin (1:1000, Sigma Aldrich), rabbit polyclonal anti-IFT22, (1:200, Sigma Aldrich), rabbit polyclonal anti-IFT25 (1:100, Thermo Scientific); rabbit poly/monoclonal anti-IFT46 (provided by Frédéric Mallein-Gerin); rabbit poly/monoclonal anti-IFT88 (provided by Chantal Desdouets); rabbit polyclonal anti-GLI1 (1:50, Abcam), goat polyclonal anti-GLI2N, (1:50, Santa Cruz) or rabbit polyclonal anti-Smoothened (1:100, Abcam) antibodies. Secondary antibodies were used as described above. Fluorescent images were obtained with a Zeiss LSM700 (Carl Zeiss SAS) laser scanning microscope.

## Results

### Phenotypes and genotypes

**Figure 1:**
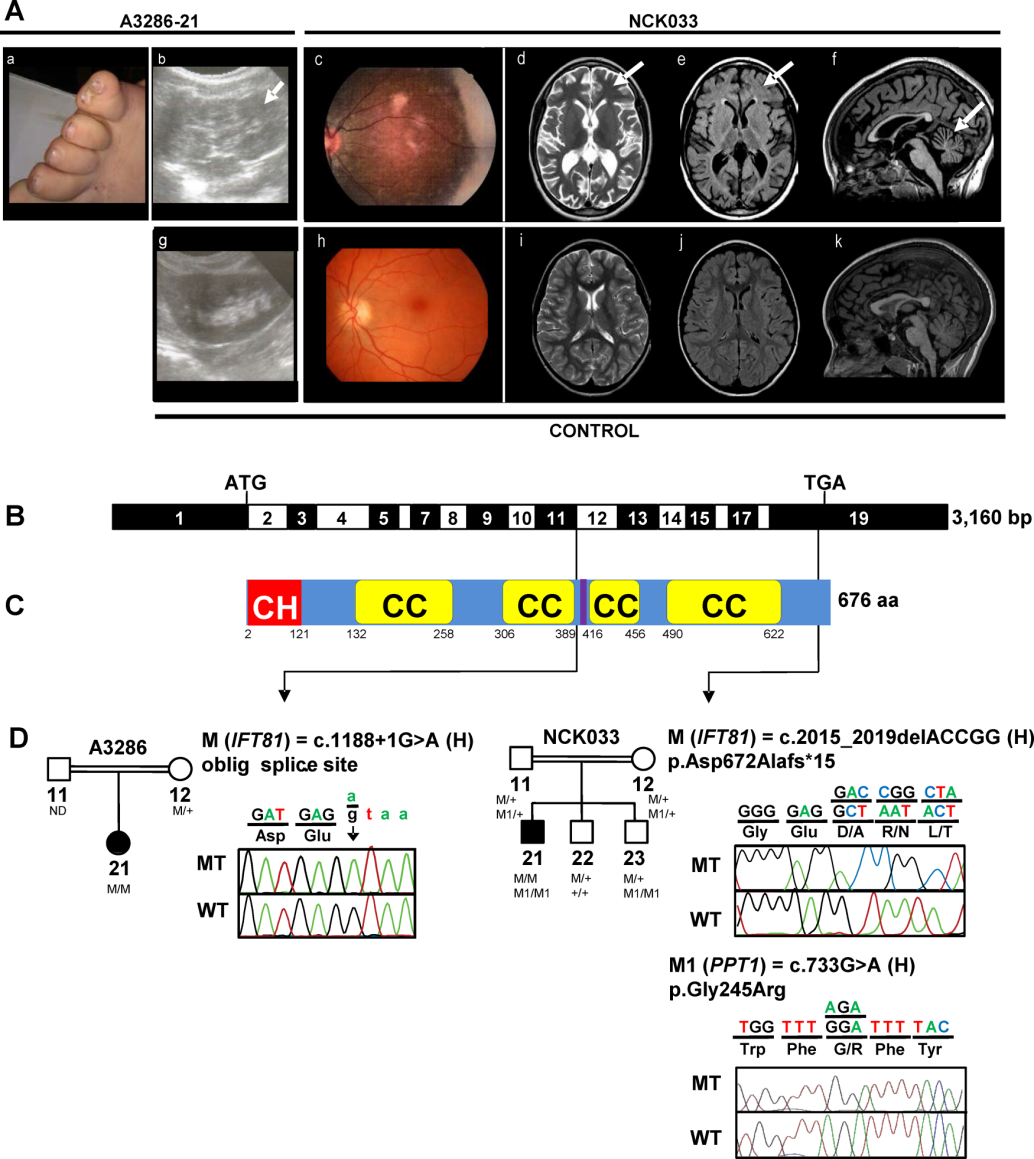
Identification of recessive mutations of *IFT81* in two consanguineous families with a ciliopathy phenotype. (A) Clinical features of A3286-21 and NCK033. (a) postaxial polydactyly of the feet (A3286-21); (b) renal sonography showing hyperechogenic kidneys with loss of corticomedullary differentiation, and small medullary cysts (arrow) (A3286-21); (c) retinophotography showing salt and pepper aspect of the fundus with mild attenuation of retinal vessels (NCK033); (d–f) Axial FSE T2, axial FLAIR and sagittal 3DT1 FSGR brain MRI weighted images showing (d and e) hyperintensities of the periventricular and subcortical white matter (arrows), and (f) cerebellar atrophy (arrow) (NCK033). (g), (h), (i), (j), (k) show control renal sonography, retinophotography and age-matched axial FSE T2, axial FLAIR and sagittal 3DT1 FSGR brain MRI weighted images, respectively. (B) Exon structure of *IFT81* cDNA (NM_014055.3). Positions of start codon (ATG) and stop codon (TGA) are indicated. (C) Domain structure of IFT81 protein. IFT81 contains an N-terminal calponin homology-domain (CH), four coiled-coil domains (CC) and a linker region for binding of IFT22/RABL5 (purple), separating the two N-terminal coiled-coils from the two C-terminal coiled-coils.^23^ ^25^ (D) Relation of two homozygous (H) mutations to exons and protein domains is indicated by black arrows. Pedigrees and chromatograms of mutant-alleles (MT) are shown above wild type controls (WT). M: mutant *IFT81* alleles; M1: mutant *PPT1* alleles. IFT, intraflagellar transport; FSE, fast spin echo; FSGR, fast spin gradient echo.

To identify disease-causing mutations, we independently applied different targeted candidate gene amplification methods with consecutive NGS to a two-centre cohort of 1628 (1056+572) individuals with ocular, neurological, skeletal and/or renal symptoms consistent with the diagnosis of ciliopathy. We first conducted a candidate gene screening of all 14 genes encoding IFT-B complex proteins (see online supplementary table S1) in 1056 individuals with NPHP-RC. In a 5-year-old girl of consanguineous Egyptian descent (A3286-21), who clinically presented with polydactyly, intellectual disability and NPHP, we detected a homozygous mutation affecting an obligatory donor splice site in *IFT81* (*intraflagellar transport 81 homologue Chlamydomonas*; RefSeq accession number: NM_014055.3, MIM 605489) (c.1188+1G>A) (table 1 and figure 1A–D). This variant was absent from all publicly available SNP databases and is predicted to be pathogenic by inframe skipping of exon 11 (−100% predicted change by MaxEnt/NNSPLICE/HSF). Familial segregation analysis was consistent with biallelism (table 1). At birth the affected individual displayed postaxial polydactyly of the feet (figure 1Aa). At age of 1.5 years, bilateral hyperechogenic kidneys with loss of corticomedullary differentiation and small medullary cysts were detected by renal ultrasound (figure 1Ab). Kidney function, however, as measured by serum creatine, was still preserved at the age of 5 years. Intellectual disability was moderate and comprised delayed speech and an IQ of 70 (∼2 SDs below the mean). Cerebral MRI was not available to assess the presence of correlating morphological brain abnormalities. Funduscopic eye examination revealed normal retinal morphology at an age of 5 years, but electroretinographic (ERG) recordings were not performed.

**Table 1:** Mutations of *IFT81* and *PPT1* in two families with a ciliopathy phenotype

Family-individual/age	Gene	Nucleotide alteration†	Deduced protein change	Exon/intron (zygosity)	Parental consanguinity	Renal disease (age of onset)	Eye disease (age of onset)	Additional clinical features
A3286-21/5 year	*IFT81*	c.1188+1G>A	5′ splice site	11 (Hom) m: het/p: ND	Yes	NPHP (1.5 year)	None	Speech delay with mild intellectual disability, polydactyly (feet)
NCK-033/9.5 year	*IFT81*	c.2015_2019del	p.Asp672Alafs*15	20 (Hom) m: het/p: het	Yes	Polyuria/Polydipsia (9.5 year)	RD (4 year)	Speech delay with mild intellectual disability, cerebellar atrophy
	*PPT1*	c.733G>A	p.Gly245Arg	8 (Hom) m: het/p: het				

†cDNA mutations are numbered according to human cDNA reference sequence NM_014055.3, isoform 1 (*IFT81*) and NM_000310.3, isoform 1 (*PPT1*), where +1 corresponds to the A of ATG start translation codon.

het, heterozygous; Hom, homozygous; IFT, intraflagellar transport; m, maternal; ND, no data; NPHP, nephronophthisis; p, paternal; RD, retinal dystrophy.

Beyond family A3286, no further biallelic variants were considered ‘disease-causing’ in the remaining cohort of 1056 patients. Although mutations in genes known to be associated with NPHP once mutated (*NPHP1-NPHP13*) had been previously excluded, we further assessed A3286-21 for competing deleterious variants by combining WER with homozygosity mapping.^13^ ^22^ In concordance with our previous finding, the *IFT81*-splice site variant (c.1188+1G>A) was ranked the most likely pathogenic allele, residing within a stretch of homozygosity on chromosome 12q (non-parametrical LOD score >2) (see online supplementary figure S1).

Second, we performed targeted resequencing of 1666 ciliary genes (‘ciliome resequencing’) in another 572 individuals with NPHP-RC, retinal or multisystemic ciliopathies. As a result, in an individual with retinal dystrophy and intellectual disability (NCK033), we found a homozygous deletion of five nucleotides in *IFT81* resulting in a loss-of-stop codon and extension of the predicted protein by 10 amino acids (c.2015_2019del (p.Asp672Alafs*15)). For complete mutation analysis, consecutive WER was performed in NCK033. Thereby, nine additional rare homozygote variants were identified (see online supplementary figure S1), including a missense change (c.733G>A (p.Gly245Arg)) in the gene *PPT1* encoding the palmitoyl-protein thioesterase (NM_000310.3; MIM600722). Familial segregation analysis demonstrated biallelism of the *IFT81* and *PPT1* changes (figure 1D and table 1). The PPT1 enzyme activity in the blood of the affected individual was strikingly decreased (5% of residual activity) confirming the clinical diagnosis of neuronal ceroid lipofuscinosis-1 (MIM 265730). None of the patients’ two brothers inherited both *IFT81* mutant alleles but one of them (NCK033, 23) inherited both *PPT1* mutant alleles (figure 1D). The affected individual (NCK033) is the first of three children of first-cousin Algerian parents (table 1 and figure 1A–D). He was born at term after unremarkable pregnancy and delivery. At an age of 4 years, the affected individual presented with visual loss, speech delay, mild intellectual disability and balance problems despite a reportedly normal cerebral MRI. Ophthalmological examination at the age of 7 years showed abnormal ocular movements, hemeralopia and poor vision. Retinography further revealed a degenerative aspect at the fundus (figure 1Ac), and ERG showed severely altered photopic and scotopic traces (see online supplementary figure S2), supporting early onset rod-cone dystrophy. Update of ophthalmological data at the age of 11 years revealed complete blindness with no residual light perception. Detailed neurological examination at age 9.5 years demonstrated an extrapyramidal and pyramidal syndrome with deep tendon and bilateral Babinski reflex. He also exhibited stereotypies, poor speech and echolalia. Repeated cerebral MRI showed diffuse cerebellar atrophy without molar tooth sign. In addition, axial flair images evidenced unusual major signal enhancements in the periventricular and subcortical white matter (figure 1Ad–f). Recently, he manifested with night enuresis, polyuria and polydipsia, but renal imaging and function still presented normal. Body measures and radiological bone examination were unremarkable.

Neither of the two *IFT81* mutations has previously been reported in SNP databases (1000 genomes, dbSNP, EVS). Moreover, inspection of *IFT81* for reported loss-of-function variants (splicing, nonsense and frameshifts) in the EVS data set revealed no homozygous but 10 heterozygous truncating variants (c.190C>T, p.Arg64*; c.297C>G, p.Tyr99*; c.365T>A, p.Leu122*; c.359_360insT, p.Leu122Phefs5*; c.648_649del2, p.Glu219Argfs41*; c.1195C>T, p.Arg399*; c.1441C>T, p.Arg481*; c.1534C>T, p.Arg512*; c.1876C>T, p.Arg626*; and c.249-1G>A), all of which had a minor allele frequency of less than 0.04% out of 13 000 alleles from individuals with European and African–American ancestry. These observations indicate that *IFT81* does not accumulate loss-of-function variants in the general population, suggesting that its disruption is consistent with causing a recessive disease.

*IFT81* consists of 19 exons (figure 1B), which encodes a 676-residue protein, containing four coiled-coil domains, and a recently characterised calponin-homology NDC80 NUF2 Calponin Homology (NN-CH) domain at its N-terminus.^23^ The NN-CH motif constitutes a tubulin-binding site required for cilia assembly and maintenance. The coiled-coil domains were shown to mediate interaction with IFT74, IFT52 and the IFT27/IFT25 complex.^24^ Very recently, a linker region between the two N-terminal and C-terminal coiled coils was mapped to constitute a binding site for the small GTPase IFT22/RABL5 (figure 1C).^25^

### Functional allele testing—decreased ciliation though unchanged ciliary localisation of IFT81

**Figure 2:**
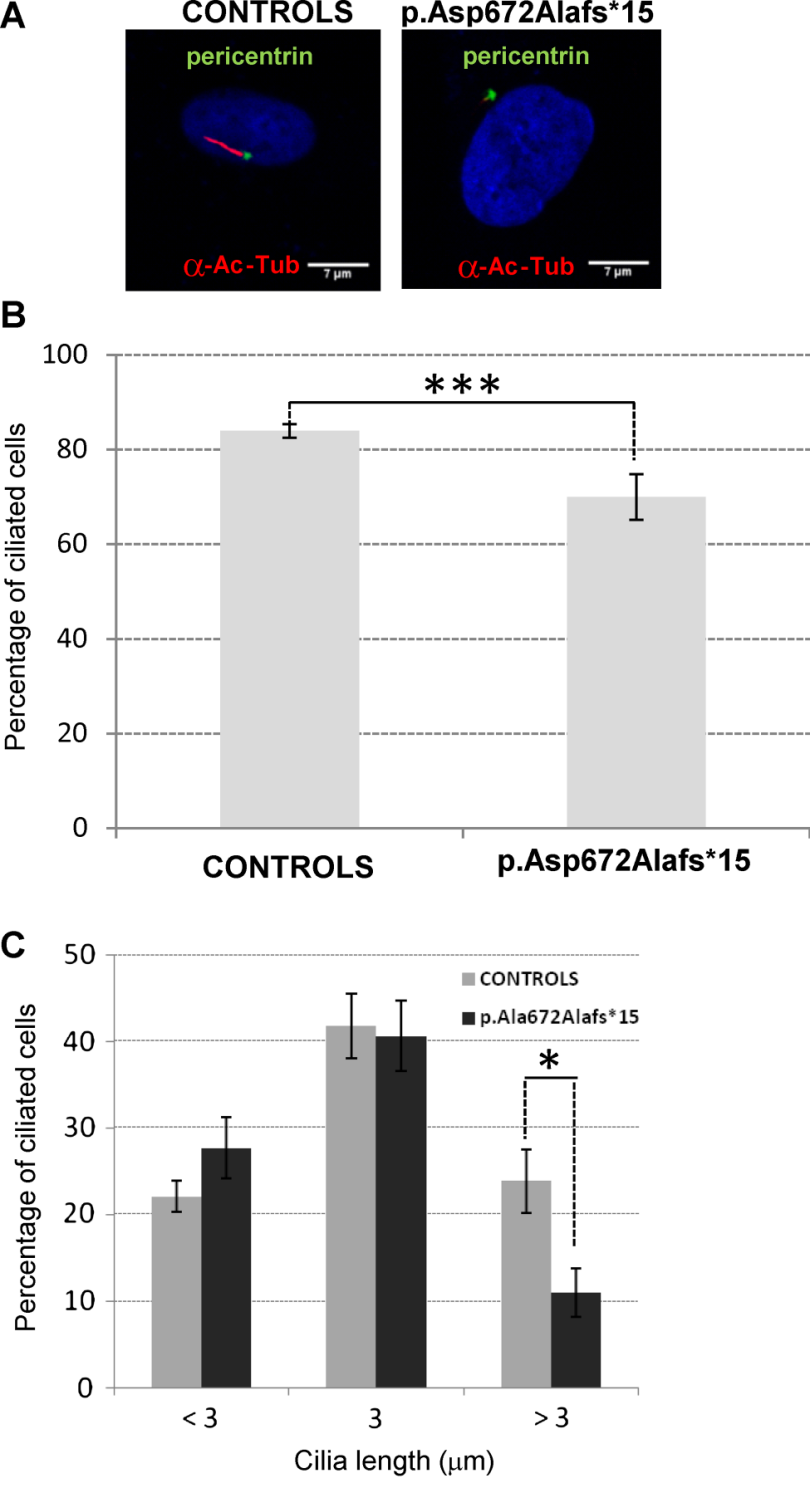
Cilia abundance and ciliary length**.** Immunofluorescence staining was performed in cultured human fibroblasts from four healthy controls and affected individual NCK033 (p.Asp672Alafs*15). (A) Cilia axonemes were stained using mouse monoclonal antiacetylated α-tubulin. (B) The mean number of ciliated cells in the sample from the affected individual is significantly decreased compared with control samples: 69.9% vs 83.9% calculated from two independent experiments (n=973 patient cells and n=895 control cells (C1: n=524; C2: n=131 and C3: n=240 cells)); ***p value=0.000026. (C) The proportion of cells with cilia length ≤3 μm is similar in the affected individual and control cell lines. Conversely, the proportion of cilia >3 μm is significantly lower in cells from the affected individual compared with the controls. *p Value=0.017 calculated from 100 patient cells and 296 control cells (C1: n=150, C2: n=78, C3: n=68).

To assess whether *IFT81* mutant alleles affect cilia abundance, morphology or the subcellular localisation of IFT81, we performed immunofluorescence microscopy in primary skin fibroblasts of individual NCK033 and 4 healthy controls upon 48 h of serum starvation. The abundance of ciliated fibroblasts was significantly reduced in cells from the affected individual compared with controls (mean mutant cells vs mean control cells: 69.9% vs 83.9%, p=0.000026; mean control cells in the 83.6%±3.6% control range),^20^ indicating that the p.Asp672Alafs*15-protein variant causes defects in ciliogenesis and/or cilia maintenance (figure 2A, B). Overall, ciliary length of mutant cells appeared smaller than that of controls further supporting this hypothesis (figure 2C).

**Figure 3:**
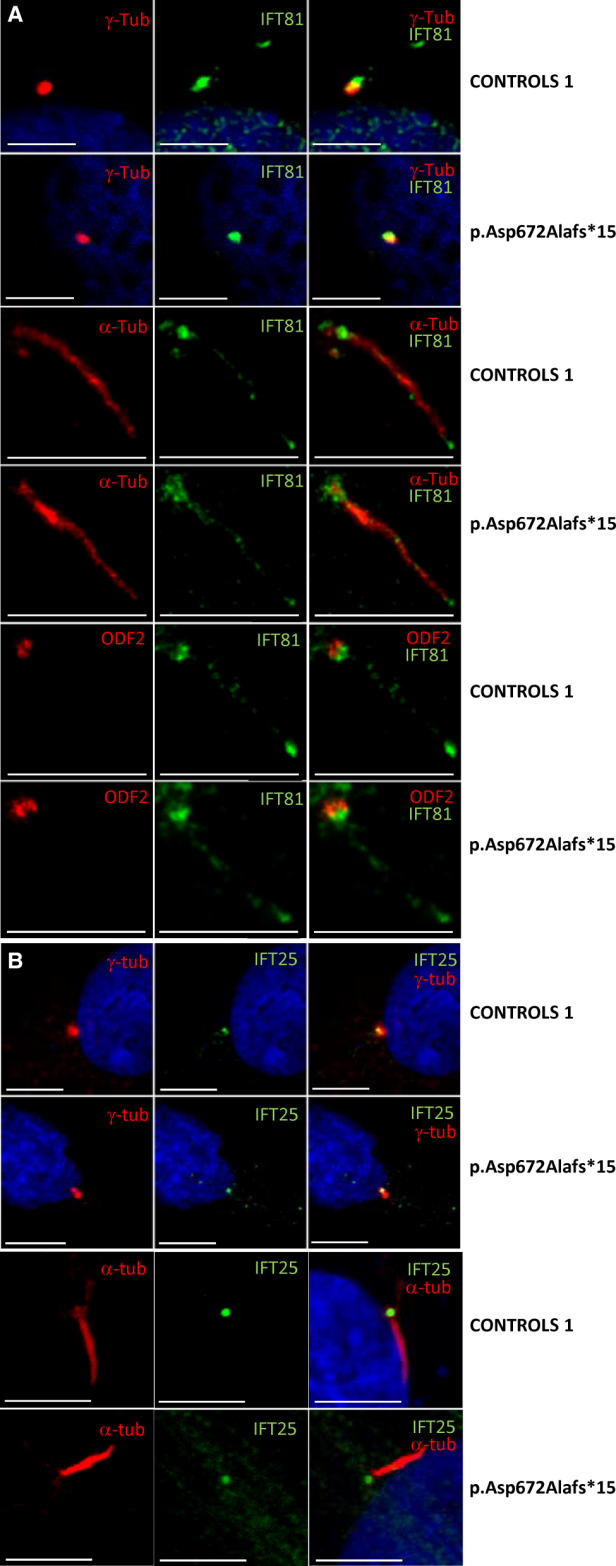
IFT81 localises to primary cilia with enrichment at base and tip. Immunofluorescence staining was performed in cultured human fibroblasts from healthy controls and affected individual NCK033 with the homozygous frameshift mutation (p.Asp672Alafs*15). (A) Note that cilia localisation of IFT81 to primary cilia did not show significant difference between control and mutant fibroblasts. (B) Ciliary signal of IFT25 did not show a significant difference between control and mutant fibroblasts. Scale bars represent 5 μm. IFT, intraflagellar transport.

In ciliated fibroblasts from the affected individual (NCK033), however, IFT81 showed no difference compared with controls in terms of protein abundance as determined by western blot analysis (see online supplementary figure S3) as well as ciliary localisation (same predominant localisation to the tip and base at a distance from the centriole) and staining intensity as determined by immunocytochemistry analysis (figure 3A). To further address the question, whether the extended IFT81 protein would lead to impaired ciliary trafficking of other IFT-B core components, we stained for the IFT81-interactors IFT25 and RABL5/IFT22.^25^ Signals of IFT25 and RABL5/IFT22 were not decreased in the mutant fibroblasts (see figure 3B and online supplementary figure S4C). Likewise, staining for IFT88 and IFT46 which form a ternary subcomplex with IFT52^26^ ^27^ was similar in the control and the fibroblasts from the affected individual (see online supplementary figure S4A, B).

### Functional allele testing—*GLI2* expression and localisation

**Figure 4:**
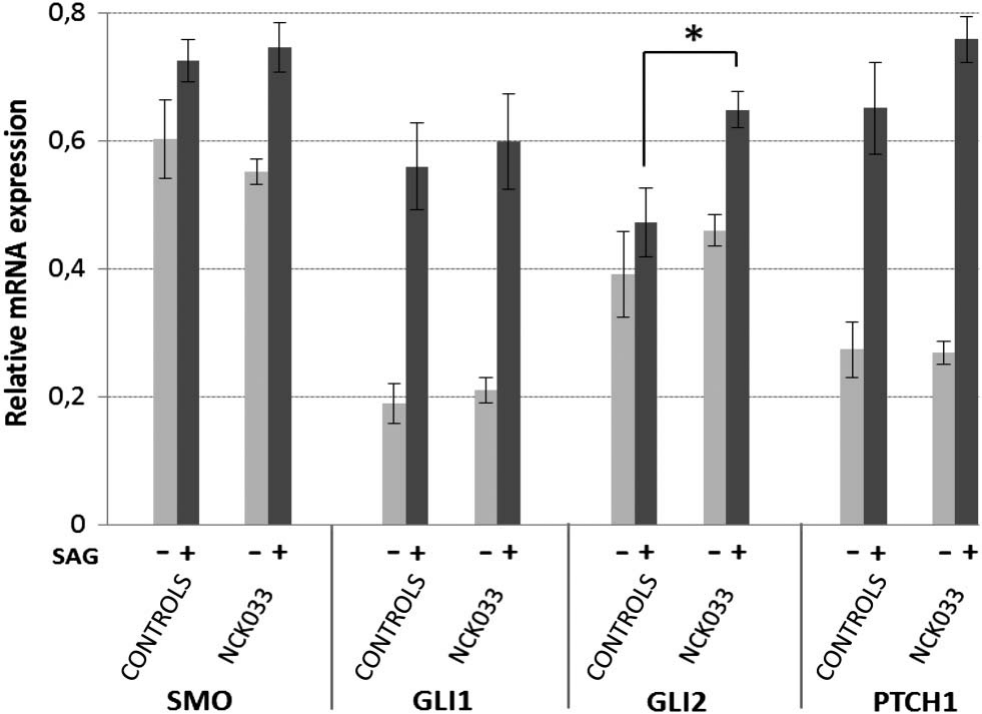
Sonic hedgehog activity in *IFT81* mutant fibroblasts (NCK033, p.Asp672Alafs*15) compared with three independent human control fibroblasts. All fibroblasts were serum-starved and maintained in culture without stimulation (−) or exposed to (+) a smoothened agonist (SAG). Relative expression levels of *SMO*, *GLI1*, *GLI2* and *PTCH1* mRNAs were determined by quantitative real-time PCR. Error bars represent the SEM derived from three independent experiments, each using triplicates. p Values were calculated applying unpaired Student's t test (*p=0.015).

The role of primary cilia and IFT in sonic hedgehog (Shh) signalling is well established.^7^ To evaluate the impact of human *IFT81* mutations on Shh signalling, we performed quantitative real-time PCR with primers specific to *GLI1* (MIM 165220), *GLI2* (MIM 165230), *PTCH1* (MIM 601309) and *SMO* (MIM 601500). Interestingly, mRNA expression of the Shh-effector *GLI2*, but none of the other pathway components was significantly increased in the *IFT81* mutant cell line (c.2015_2019del) compared with three different controls, when stimulated with a smoothened agonist for 24 h (SAG, 100 nM) (figure 4). We subsequently analysed the endogenous subcellular localisation of these Shh pathway components. By immunostaining, however, no major differences in ciliary localisation of SMO, GLI1 and GLI2 were detected before and after SAG stimulation in control and patient fibroblasts (see online supplementary figure S5).

## Discussion

Here, we report the identification of human mutations of *IFT81* in two unrelated individuals with multisystemic symptoms consistent with ciliopathy phenotypes. Unlike many other IFT encoding genes, no mouse model has been reported for *Ift81*. However, an *ift81* zebrafish mutant (*hi409/larry*) was first identified in a genetic screen for fish with cystic kidney disease.^28^ Somite-stage gene expression showed enrichment along classically ciliated organs; notochord, otic vesicle, pronephric duct, and around the cerebral ventricles, pointing towards an essential role of IFT81 in embryonic development of kidneys and brain.^28^ This is consistent with the view that *IFT81* mutations can cause a ciliopathy in humans.

Mechanistically, our findings indicate altered Shh-signalling for at least one of our alleles (c.2015_2019del) (figure 4). The importance of GLI2 and the hedgehog pathway for limb development in mammals is well established as postaxial polydactyly is a common feature in individuals with *GLI2* mutations^29^ and aberrant Shh-signalling has long been shown to result in clinical abnormalities of digit number and identity.^30^ Correct anterior-posterior digit patterning underlies secretion of Shh by posterior mesenchymal cells in the zone of polarising activity.^31^ Digital malformations in IFT mutant mice were observed in states of hypoactive (*wimple*, *polaris*) and hyperactive (*alien*, *sopb*) Shh-signalling, indicating that different mechanisms lead to disruption of physiological pathway function.^7^ ^8^ ^32–34^ As fibroblasts of A3286-21 were not available for our study, we have to speculate that defective GLI2 and Shh-signalling, might also be a mechanistic reason for postaxial polydactyly and intellectual deficits in the respective patient, carrying the obligatory splice-site mutation. On the other hand, absence of skeletal defects in patient NCK033 suggests that the modification of the C-terminus of IFT81 due to the c.2015_2019del mutation might alter hedgehog signalling during brain patterning but not during skeletal development. Furthermore, genetic modifiers may have contributed to these phenotypical variations. In the mouse *Gli2* has been shown to be required for the full extent of growth and elaboration of the cerebellar lobes.^35^ In addition, truncating *GLI2* mutations have been reported to cause holoprosencephaly with and without pituitary hormone deficiencies and craniofacial features.^29^ The alteration of GLI2 expression in fibroblasts from NCK033 is consistent with the cerebellar atrophy in this individual. Cilia of mutant mice and human fetal samples have revealed a pivotal role of hedgehog signalling for the proliferation of granule cell progenitors. In addition, a direct correlation between Shh-signalling disruption, defective granule cell progenitor proliferation, and cerebellar hypo/aplasia in individuals with Joubert and Meckel syndrome has been described.^34^ ^36^ While the cerebellar involvement is consistent with the cilia defect, the major signal enhancements in the periventricular and subcortical white matter (figure 1Ad–f) is not usually seen in patients with ciliopathies. From this point of view, it is important to stress that in addition to defective ciliogenesis, the activity of the PPT1 enzyme in the blood of NCK033 was dramatically low. PPT1 enzyme deficiencies are known to cause neuronal ceroid lipofuscinosis-1 (MIM 265730). This fatal neurological condition includes early onset severe retinal dystrophy and marked brain lesions. Importantly, segregation analysis of *IFT81* and *PPT1* variants showed that the younger of the two brothers is homozygous for *PPT1* but heterozygous for the *IFT81* mutation (figure 1D). While the index patient, NCK033, manifested at 4 years with visual loss, speech delay and ataxia, his 4-year-old brother currently has no overt retinal or neurological problems. In the absence of ERG and brain MRI from NCK033's non-affected brother, a clinical follow-up will help to clearly discern the respective roles of *PPT1* and *IFT81* defects in the disease expression.

Considering kidney involvement, only the younger of the two individuals (A3286-21) had structural lesions, albeit with preserved renal function. However, the age of onset of renal manifestation has been shown to vary considerably between and within genetic subtypes of renal ciliopathies.^37^ Thus, it is difficult to anticipate whether the second individual (NCK033), age 10 years, is at risk for developing renal failure or whether the mutation extending the C-terminus of IFT81 does not affect kidney function. Recently he manifested symptoms of night enuresis, polyuria and polydipsia which might either be due to the neurological dysfunction or constitute first signs of a structural kidney disease.^38^ Similarly, retinal dystrophy in individual NCK033 only manifested at age 4 years, thus leaving it open whether the younger individual (A3286-21) may develop retinal dystrophy later in life. In addition, retinal involvement in individuals with other IFT mutations was also shown to be quite variable and is difficult to diagnose at an early stage without available ERG recordings, such as in A3286-21.^15^

In summary, we here identify mutations in *IFT81* and suggest that they represent an exceedingly rare cause of a ciliopathy phenotype in humans. The relatively mild clinical presentation together with insights from functional analysis indicates that both alleles constitute hypomorphs. IFT81 contains coiled-coil domains mediating its interaction with IFT74, and an N-terminal NN-CH domain, which is part of a tubulin-binding module. We suspect that neither of the detected human mutations would disrupt the IFT81-IFT74 complex.^23^ The interaction with IFT22/RABL5, however, was mapped to the linker region of IFT81 that localises just the C-terminal of the region encoded by exon 11 that is presumably skipped in A3286-21 (figure 1C, D).^25^ This may result in alteration or even abrogation of physical interaction between IFT22/RABL5 and IFT81. Extension of IFT81 by 10 amino acids (NCK033) does not lead to mislocalisation of IFT81 within the cilium, but to reduced ciliation, and alteration of Shh-signalling (figure 4), thereby possibly explaining some of the brain malformation. In contrast to other IFT-related phenotypes, major skeletal abnormalities were absent in the two individuals with *IFT81* mutations. The low number of identified individuals and the additional *PPT1* mutation in one of them, however, makes it difficult to draw genotype-phenotype correlations. As IFT81 represents the hub of the IFT-B core, more severe IFT81 defects may only be found in disease phenotypes that are incompatible with life.

## Supplementary Material

Web supplement
