# Supplementary material for: IFT81, encoding an IFT-B core protein, as a very rare cause of a ciliopathy phenotype
Source: J Med Genet. 2015 Aug 14;52(10):657–65. doi: 10.1136/jmedgenet-2014-102838 (PMC4621372; doi:10.1136/jmedgenet-2014-102838)
Supplement: Web supplement [file jmedgenet-2014-102838-s1.pdf]

Figure S1

A

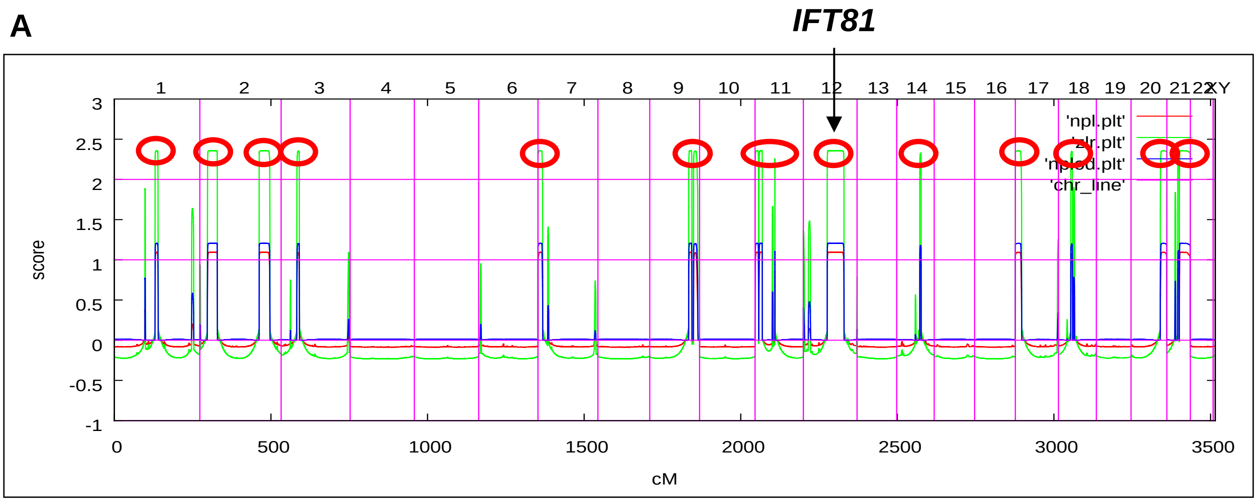

B

Individual	Gene	hg19 pos.	Accession #	Nt change >	AA change p.	Amino acid conservation							Poly 2	Mut Taster	SIFT	1000 genomes'	EVS
						Mm	Gg	Xt	Dr	Ci	Ce	Dm					
A3286-21	*IFT81*	chr12:110600871	NM_014055.3	G>A	5' splice site	Nucleotide is 100% conserved										no	n/a
	GDAP2	chr1:118420677	NM_017686	G>C	P467R	P	P	P	P	-	-	K	1	DC	Tol	no	n/a
	ADAM30	chr1:120438619	NM_021794	C>T	G114D	G	G	G	G	G	G	G	0,999	Tol	Del	no	T=1/C=13005
	WDR35	chr2:20137642	NM_001006657	C>T	R721H	R	R	R	R	R	K	R	0,015	DC	Tol	no	T=1/C=13003
	XDH	chr2:31565143	NM_000379	C>T	S1142N	S	N	D	D	D	D	H	0	Tol	Tol	no	n/a
	LAMC3	chr9:133946985	NM_006059	G>A	V1062I	V	G	L	A	G	V	L	0,001	Tol	Tol	rs150681325	A=2/G=13004
	OAS2	chr12:113448219	NM_016817	C>T	P697L	P	-					K	0,622	Tol	Tol	no	n/a
	DPH1	chr17:1936837	NM_001383	C>G	P39A	P	E	-	E	D	E	Q	0	Tol	Tol	no	n/a
NCK033	*IFT81*	chr12:110656015	NM_014055.3	delACCGG	D672Afs*	DRLVL	DRLIL	DRLVL	?	?	-	?				no	n/a
	PPT1	chr1:40542579	NM_000310.3	G>A	G245R	G	G	G	G	G	G	Q	1	-	-	no	n/a
	MUTYH	chr1:45331783_45331784	NM_012222.2	insGGAGCCCTGGGACC	V352Afs*58	-	-	-	-	-	-	-	-	-	-	no	n/a
	NHS	chrX:17375984	NM_198270.2	C>T	A76V	A	-	-	-	-	-	-	0,039	-	-	no	n/a
	SEPT6	chrX:118771080	NM_145799.3	A>G	Q289R	Q	Q	Q	Q	Q	R	Q	0,997	DC	Tol	no	n/a
	CSMT1	chr16:1470712	NM_001272051.1	T>G	L5V	L	-	T	F	-	-	-	0,476	-	Tol	no	n/a
	CC154	chr16:1493840	NM_001143980.1	C>T	P61S	P	P	P	P	-	-	-	0,903	-	Tol	no	n/a
	NUAK1	chr12:106461470	NM_014840.2	C>T	R366W	R	R	-	-	-	-	-	0,999	-	Tol	no	n/a
	OTUD7A	chr15:31776211_31776212	NM_130901.1	insGCCGCCGCCGCC	p.A689_Thr690insProProPro	-	-	-	-	-	-	-	-	-	-	no	n/a

Fig. S1: Homozygosity mapping of A3286-21 and final WER-variant table for A3286-21 and NCK033. (A) nonparametric LOD (NPL) scores from whole-genome mapping are plotted across the human genome. The x-axis shows Affymetrix 250K Styl array SNP positions on human chromosomes concatenated from pter to qter. Genetic distance is given in cM. Thirteen maximum NPL peaks (red circles) indicate candidate regions of homozygosity by descent. Note that the locus of *IFT81* residues within one of the homozygous regions on chromosome 12 (black arrow). **(B)** Final remaining variants after filtering process. *IFT81* variants are highlighted in yellow to indicate ranking as most likely disease causing. *WDR35*-missense variant in A3286-21 is highlighted in grey to indicate that nucleotide T is present in several closely related mammals (squirrel monkey, marmoset, dog, elephant) pointing towards a benign polymorphism. In NCK033, in addition to the change in *IFT81*, the *PPT1*-variant is most likely disease causing, as defects in the encoding enzyme are known to be associated with neuronal ceroid lipofuscinosis (CLN1).

Mm - Mus musculus, Gg - Gallus gallus, Xt - Xenopus tropicalis, Dr - Danio rerio, Ci - Ciona intestinalis, Ce - Caenorhabditis elegans, Dm - Drosophila melanogaster , DC - Disease causing, Tol –Tolerated, n/a – not annotated, Poly 2, Polyphen2-Humvar; Mut Taster, Mutation Taster; EVS, Exome variant server.

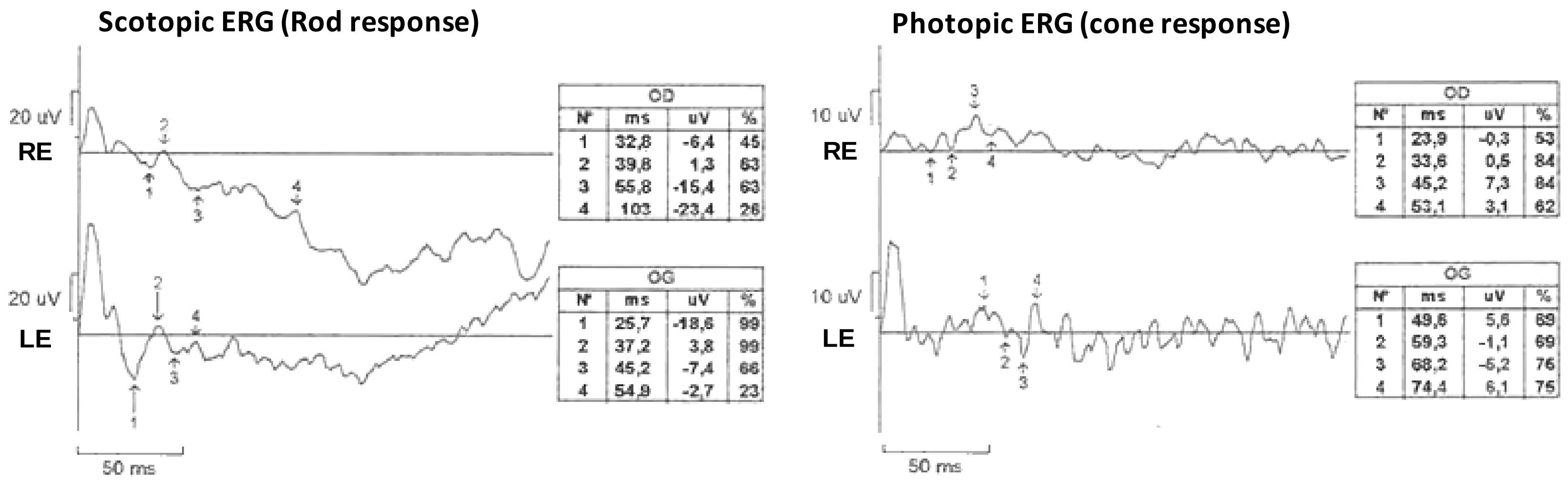

Scotopic ERG, OD (RE)

N°	ms	uV	%
1	32,8	-6,4	45
2	39,8	1,3	63
3	55,8	-15,4	63
4	103	-23,4	26

Scotopic ERG, OG (LE)

N°	ms	uV	%
1	25,7	-18,6	99
2	37,2	3,8	99
3	45,2	-7,4	66
4	54,9	-2,7	23

Photopic ERG, OD (RE)

N°	ms	uV	%
1	23,9	-0,3	53
2	33,6	0,5	84
3	45,2	7,3	84
4	53,1	3,1	62

Photopic ERG, OG (LE)

N°	ms	uV	%
1	49,6	5,6	69
2	59,3	-1,1	69
3	68,2	-6,2	76
4	74,4	6,1	75

Figure S2. Patient NCK033 scotopic and photopic ERG traces. The morphology and culminating time of rod and cone responses are normal but the maximum response amplitudes are strikingly reduced. These traces are consistent with the diagnostic of rod-cone dystrophy. RE: right eye; LE: Left eye.

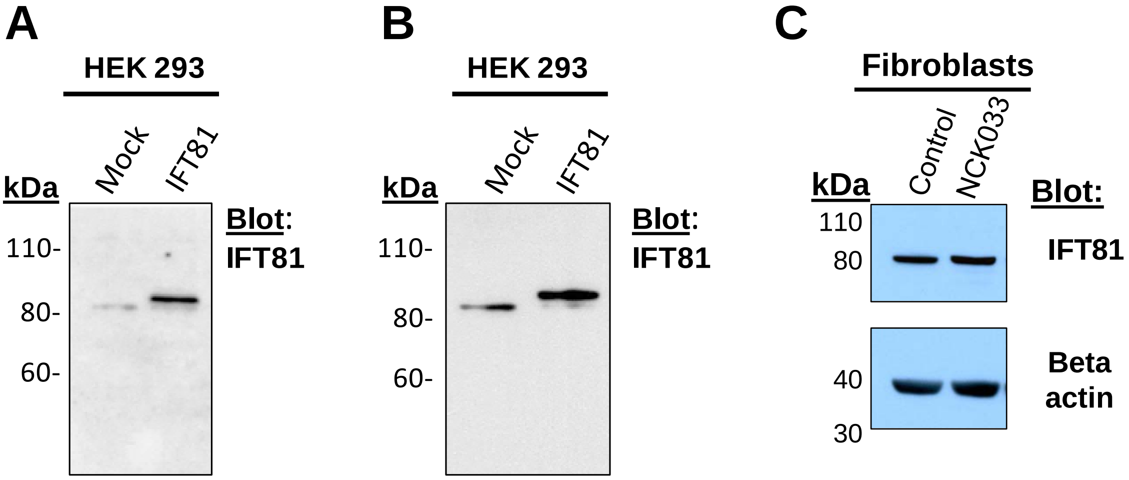

Figure S3. AB-specificity of IFT81 and protein expression in mutant fibroblasts.
(A) Overexpression of either Mock or N-terminally His/Flag tagged IFT81. Note that the N-terminal tags add 18 kDa for an expected size of 99 kDa. Overexpressed IFT81 is significantly stronger than endogenously expressed detected by the anti-IFT81 Proteintech antibody used for IF. **(B)** Overexpression of either Mock or N-terminally His/Flag tagged IFT81. Note that the N-terminal tags add 18 kDa for an expected size of 99 kDa. Overexpressed IFT81 is significantly stronger than endogenously expressed (Atlas Antibodies: HPA019087). **(C)** IFT81 is specifically detected using anti-IFT81 (Atlas Antibodies: HPA019087).

HEK293 cells transfected with Mock or N-terminally His/Flag tagged IFT81, and patient and control fibroblasts were harvested and lysed in a RIPA buffer (Sigma Aldrich) containing complete protease inhibitor cocktail (Roche, Boulogne-Billancourt, France) on ice for 30 minutes with repeated mixing. Released DNA was fragmented by 20 seconds of Ultra-turrax homogenizer (Ika-Werke, Staufen, Germany) and the lysates were centrifuged (15,000g at 4 C for 10 minutes). Proteins (50 µg) were denatured at 90 C for 10 minutes in 4X premixed protein sample buffer (XT sample Buffer; Bio-Rad) and separated by electrophoresis (50 V for 30 minutes followed by 140 V for 90 minutes at room temperature) on Mini protean TGX 4-15% (Bio-Rad). Proteins were transferred (Trans-BlotR Bio-Rad) to PVDF membranes (Bio-Rad). Membranes were blocked with phosphate-buffered saline (PBS) 0.5% Tween-20/5% dry milk powder and incubated overnight at 4 C under agitation with anti-IFT81, rabbit, polyclonal (Proteintech) 1:2000 dilutions. Membranes were washed three times in PBS 0.5% Tween-20 solution and incubated for 1 hour at room temperature with HRP-conjugated donkey anti-rabbit (Amersham GE Healthcare, Courtaboeuf, France) in 1:10,000 dilutions. ECL Western Blotting Detection Reagents (Amersham GE Healthcare) was applied according to the manufacturer's instructions and the blot was exposed to Chemi DocTM (Bio-Rad).

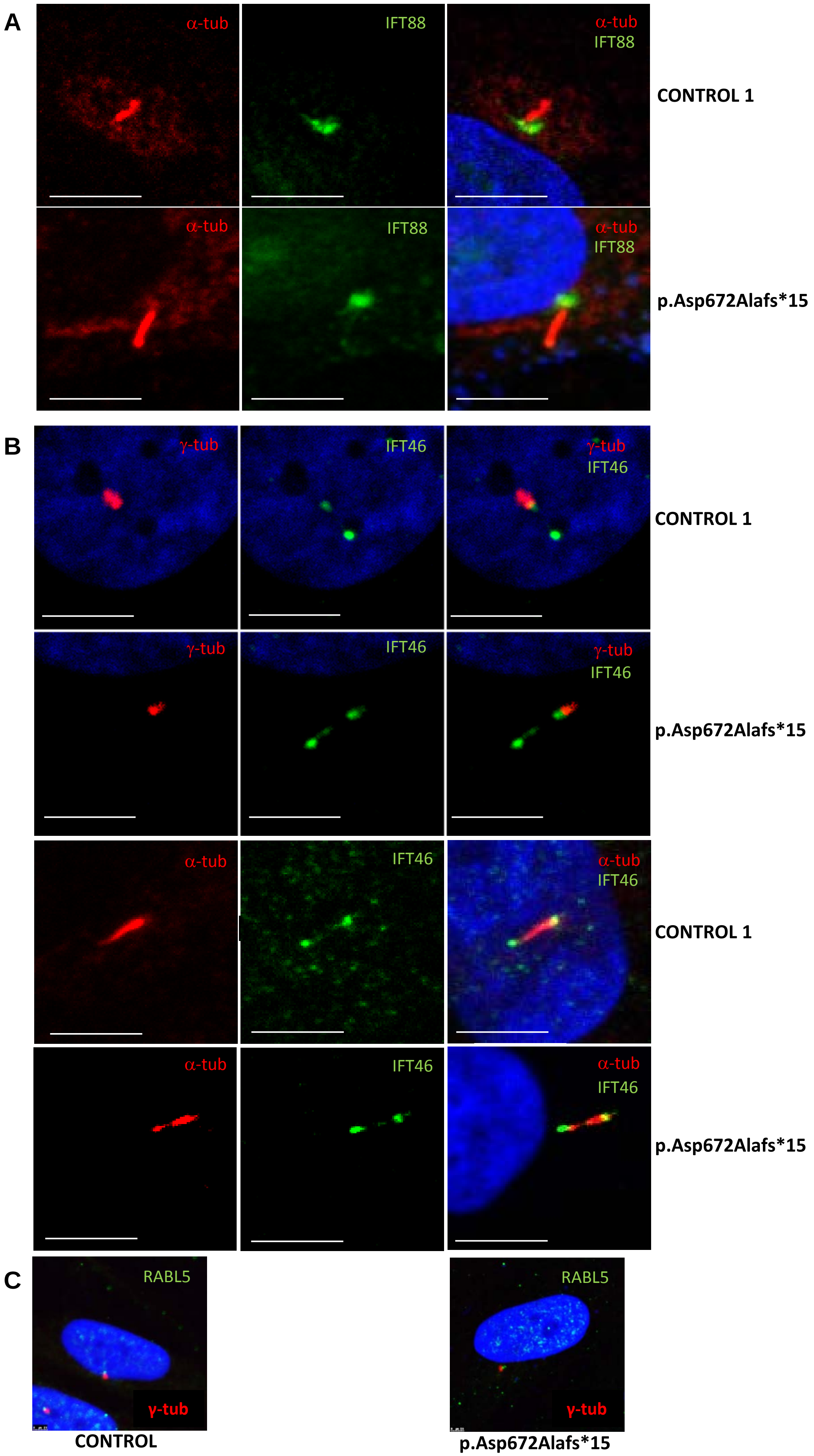

Figure S4. Ciliary staining of three additional IFT-B core components. Immuno-fluorescence staining was performed in cultured human fibroblasts from healthy controls and patient NCK033 (p.Asp672Alafs*15) after 48 hours of serum starvation. Note that ciliary localization of IFT88 **(A)**, IFT46 **(B)**, and RABL5/IFT22 **(C)** did not show significant difference between control and mutant fibroblasts.

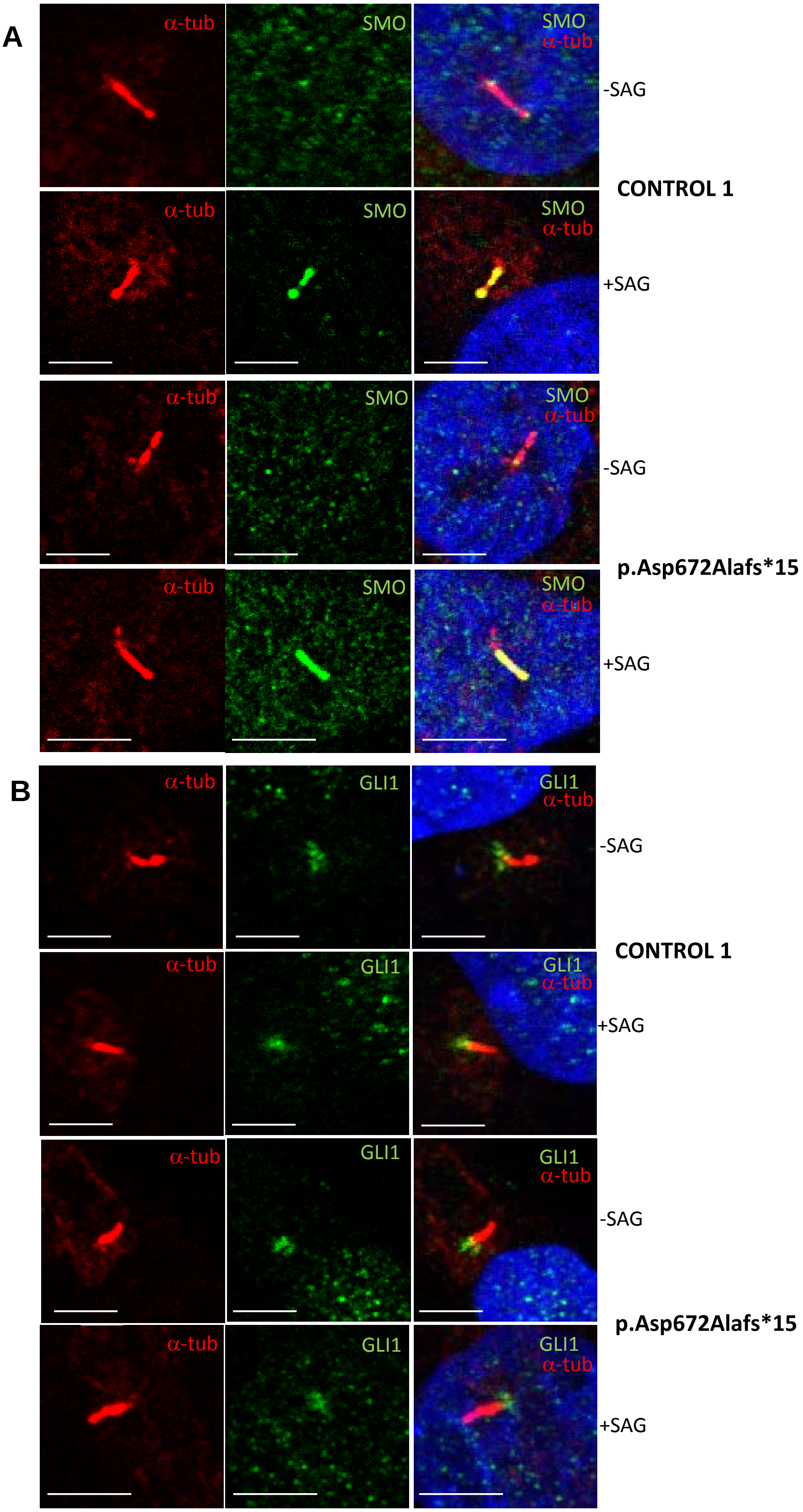

A
α-tub
SMO
SMO α-tub
-SAG
CONTROL 1
+SAG
-SAG
p.Asp672Alafs*15
+SAG
B
α-tub
GLI1
GLI1 α-tub
-SAG
CONTROL 1
+SAG
-SAG
p.Asp672Alafs*15
+SAG

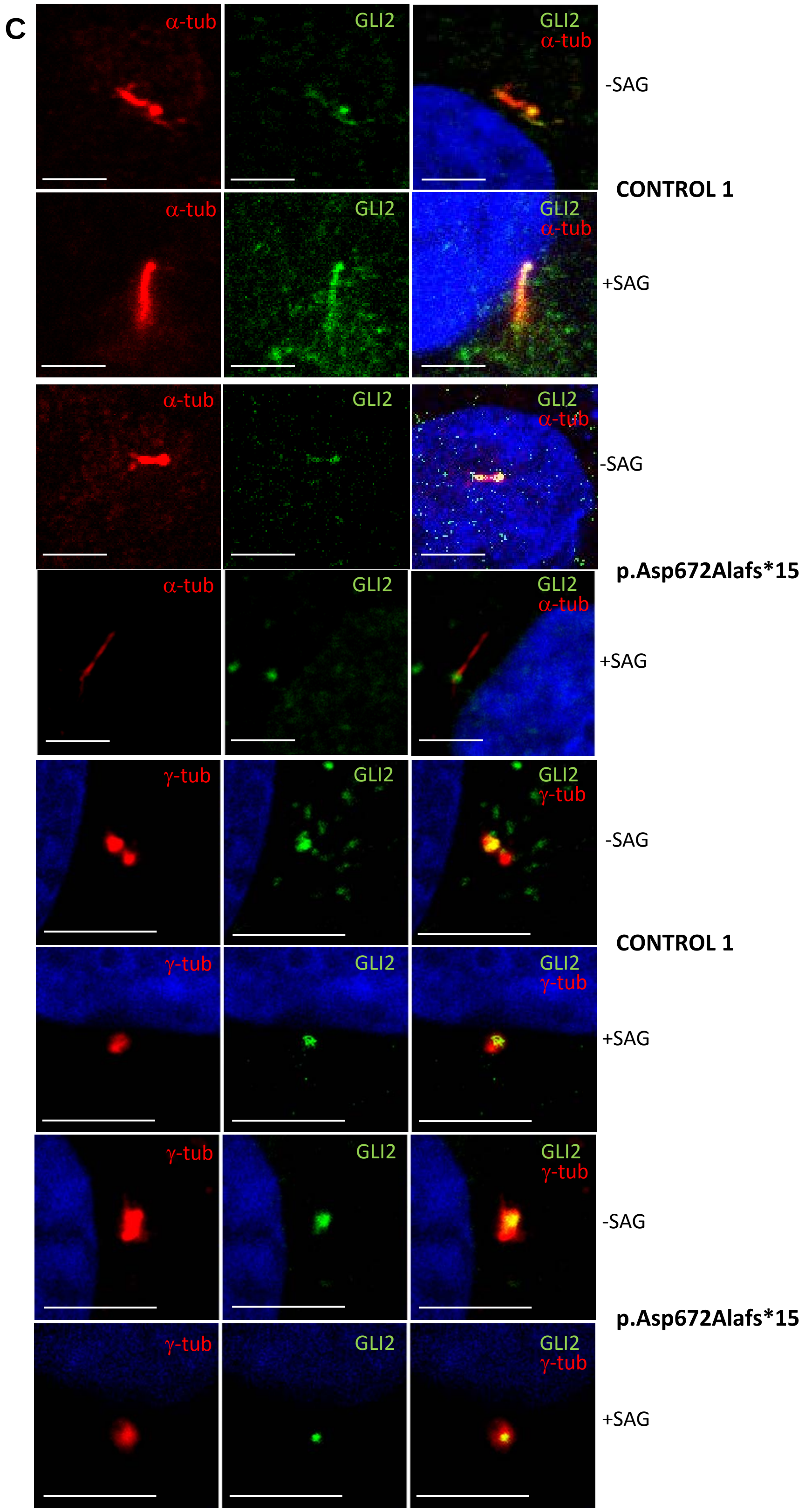

Figure S5. Localization of Shh-pathway components in *IFT81* mutant fibroblasts (NCK033: p.Asp672Alafs*15) compared to human control fibroblasts. Immunofluorescence staining was performed in cultured human fibroblasts from healthy control and affected individual (p.Asp672Alafs*15) after 48 hours of serum starvation with or without smoothened agonist for 24 hours (SAG,100nM, Santa Cruz). Cilia were stained using mouse monoclonal anti acetylated alpha tubulin, anti gamma tubulin, and either anti-SMO, anti-GLI1, or anti-GLI2 antibodies.

(A) Immunostaining show that SMO is evenly distributed under SAG activation both in the control and affected individual cells. **(B)** Immunostaining demonstrate unchanged localization of GLI1 with and without SAG in both cell lines. **(C)** Immunostaining evidence no ciliary localization change of GLI2 in the affected individual compared to the control cells. Scale bar represents 2mm.

Table S1. Fourteen genes, encoding IFT-B components, included in mutation analysis in 1,056 individuals with NPHP-RC at University of Michigan / Boston Children's Hospital. B-Core proteins are highlighted in grey.

No	*Gene symbol*	Gene name	Accession	Locus	# Exons
1	***IFT172/SLB***	intraflagellar transport 172 homolog (Chlamydomonas)	NM_015662.1	chr2:27,667,241-27,712,571	48
2	***IFT88***	intraflagellar transport 88 homolog (Chlamydomonas)	NM_175605.3	chr13:21,141,208-21,265,576	26
3	***IFT80***	intraflagellar transport 80 homolog (Chlamydomonas)	NM_020800.2	chr3:159,976,255-160,102,434	19
4	***IFT46***	intraflagellar transport 46 homolog (Chlamydomonas)	NM_020153.3	chr11:118,415,243-118,436,791	11
5	***IFT52***	intraflagellar transport 52 homolog (Chlamydomonas)	NM_016004.2	chr20:42,219,579-42,275,861	14
6	***IFT57/HIPPI***	intraflagellar transport 57 homolog (Chlamydomonas)	NM_018010.3	chr3:107,879,659-107,941,417	11
7	***IFT74/CCDC2***	intraflagellar transport 74 homolog (Chlamydomonas)	NM_001099222.1	chr9:26,956,371-27,062,931	19
8	***IFT81/CDV1***	intraflagellar transport 81 homolog (Chlamydomonas)	NM_001143779.1	chr12:110,562,140-110,656,600	18
9	***RABL5/IFT22***	RAB, member RAS oncogene family-like 5	NM_022777.2	chr7:100,956,648-100,965,093	5
10	***TRAF3IP1/IFT54***	TNF receptor-associated factor 3 interacting protein 1	NM_015650.3	chr2:239,229,185-239,309,541	17
11	***HSPB11/IFT25***	heat shock protein family B (small), member 11	NM_016126.2	chr1:54,387,234-54,411,288	4
12	***IFT20***	intraflagellar transport 20 homolog	NM_174887.2	chr17:26,655,353-26,662,495	5
13	***IFT27***	intraflagellar transport 27 homolog	NM_001177701.2	chr22:37,154,246-37,172,172	7
14	***TTC30B/IFT70***	tetratricopeptide repeat domain 30B	NM_152517.2	chr2:178,414,886-178,417,524	1

Table S2. *GLI1* , *GLI2* , *SMO* , and *PTCH1* specific primers sequences for qRT-PCR.

GENE	PRIMER SEQUENCE	ACCESSION #	LENGTH
PTCH1 :	F : GCTACTTACTCATGCTCGCC R : TCCGATCAATGAGCACAGGC	NM_001083605	140
SMO :	F : GGGAGGCTACTTCCTCATCC R : TGGTCTCGTTGATCTTGCTG	NM_005631	103
GLI1 :	F : AGAGGGTGCCATGAAGCCAC R: AAGGTCCCTCGTCCAAGCTG	NM_005269.2	161
GLI2 :	F : TGACACCAACCAGAACAAGC R : GTCCAGACACCTGGCCCTG	NM_005270	156
